# Light-Programmable Assemblies of Isotropic Micromotors

**DOI:** 10.34133/2022/9816562

**Published:** 2022-07-06

**Authors:** Shengping Che, Jianhua Zhang, Fangzhi Mou, Xia Guo, Joshua E. Kauffman, Ayusman Sen, Jianguo Guan

**Affiliations:** ^1^State Key Laboratory of Advanced Technology for Materials Synthesis and Processing, International School of Materials Science and Engineering, Wuhan University of Technology, 122 Luoshi Road, Wuhan 430070, China; ^2^Department of Chemistry, The Pennsylvania State University, University Park, PA 16802, USA

## Abstract

“Life-like” nonequilibrium assemblies are of increasing significance, but suffering from limited steerability as they are generally based on micro/nanomotors with inherent asymmetry in chemical composition or geometry, of which the vigorous random Brownian rotations disturb the local interactions. Here, we demonstrate that isotropic photocatalytic micromotors, due to the persistent phoretic flow from the illuminated to shadowed side irrespective of their Brownian rotations, experience light-programmable local interactions (reversibly from attraction to repulsion and/or alignment) depending on the direction of the incident lights. Thus, they can be organized into a variety of tunable nonequilibrium assemblies, such as apolar solids (i.e., immobile colloidal crystal), polar liquids (i.e., phototactic colloidal stream), and polar solids (i.e., phototactic colloidal crystal), which can further be “cut” into a predesigned pattern by utilizing the switching motor-motor interactions at superimposed-light edges. This work facilitates the development of active matters and motile functional microdevices.

## 1. Introduction

Self-assembly, which occurs at various scales from the growth of atomic crystals to the formation of galaxies, is a promising approach to creating new structures and materials [1]. It usually develops in thermal equilibrium and generates ordered structures determined by local or global energy minimum [2–10]. On the other hand, a number of living organisms, such as swarming microorganisms, shoaling fish, and flocking birds, can collectively self-organize into dynamic patterns out of equilibrium and thus show excellent self-adaptivity to environmental cues, such as local landscapes, food sources, and predation pressure [11, 12]. Inspired by the self-organization of living organisms, researchers are devoted to creating synthetic “life-like” nonequilibrium assemblies, which are of immense scientific and technological interest in multiple disciplines, such as artificial intelligence, active materials, and swarming robotics [12, 13].

As counterparts of living microorganisms in nature, synthetic micro/nanomotors (MNMs) are capable of autonomously moving by harvesting energy from surrounding chemicals or external fields [14–24]. Utilizing their self-propulsion and nonequilibrium interactions [15–20, 25, 26], the MNMs may form “life-like” nonequilibrium swarms (in a liquid-like state) and assemblies (in a solid-like ordered state) with emerging properties that are not found in equilibrium ones, such as dynamic clustering, reconfigurations, and self-adaptivity [27–40]. However, the so-far-developed nonequilibrium assemblies are mainly based on asymmetric MNMs (e.g., Janus microspheres) [37–40], which possess inherent asymmetry in chemical composition or geometry along their axis or orientation. These asymmetric MNMs, during self-organization, inevitably suffer from random Brownian rotations and constantly changing orientations [41], leading to randomized propulsion forces and disturbing local interactions with their neighbors. This makes the nonequilibrium interactions among them (e.g., phoretic interaction) nonuniform and difficult to control [42]. Consequently, the organized assemblies manifest limited collective states (e.g., switched only between assembly and disassembly) [43].

Herein, we report that isotropic semiconductor micromotors (MMs) experience light-direction-adaptive local interactions, and thus can self-organize into nonequilibrium assemblies with light-programmable collective positional and orientational orders. Taking isotropic micromotors of Pt-nanoparticle-decorated TiO_2_ microspheres (TiO_2_@Pt MMs) as an example, the local interactions can be reversibly switched between attraction and repulsion/self-alignment by simply changing the incident UV-light direction. Thus, the TiO_2_@Pt MMs can be frozen into apolar solids (i.e., immobile colloidal crystals) under vertical upward UV irradiation, and rapidly transformed into polar liquids (i.e., “phototactic streams”) when the direction of the UV irradiation becomes oblique downward. Under the superimposed UV irradiation from the above two directions, they can further self-organize into polar solids (i.e., “phototactic colloidal crystals”) and show dynamic on-the-fly phase transitions depending on the temporal control of the “on-off” state and light intensity (*I*) of the constituent UV lights. In addition, the nonequilibrium assemblies can also be engineered into a desired pattern by using a “top-down” light-trimming strategy based on the spatial control of the superimposed irradiation. The fluid-pressure-induced propulsion and mutual interactions of the TiO_2_@Pt MMs residing near a glass substrate are revealed by a numerical model of the semibulk phoretic flows in the electrical double layer (EDL), rather than the traditional surface-slip model [44, 45]. This work may inspire the development of novel colloidal model systems for the fundamental studies of phase transitions and the on-site assembly of intelligent functional microdevices, such as reconfigurable swarming micro/nanorobots, motile responsive photonic crystals, dynamic motile microlens arrays, and adaptive motile plasmonic devices.

## 2. Results

**Figure 1:**
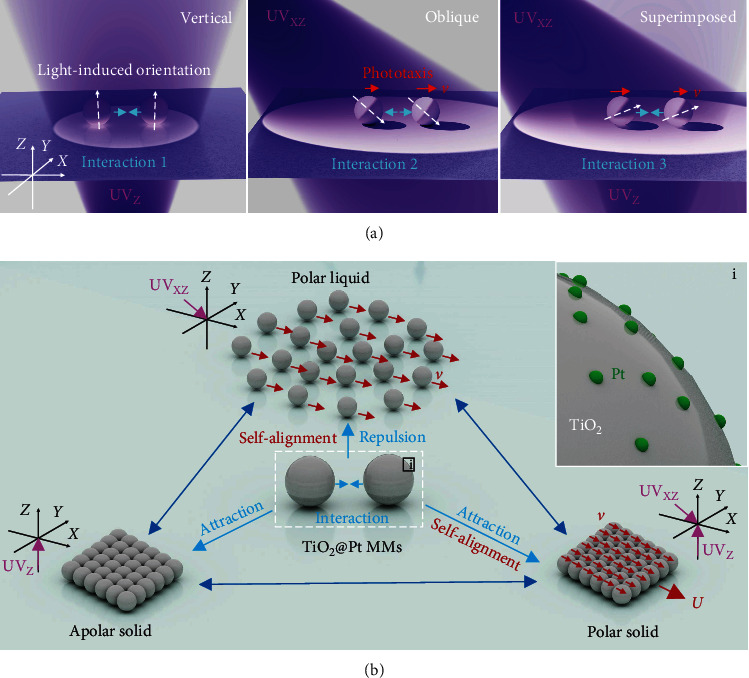
Schematic illustration of the light-programmable nonequilibrium assemblies of isotropic micromotors (MMs). (a) Light-induced uniform orientations and local orientation-dependent interactions (Interactions 1-3) of two neighboring isotropic MMs under vertical upward (UV_Z_, left panel), oblique downward (UV_XZ_, middle panel), and superimposed (UV_Z_ and UV_XZ_, right panel) UV irradiations. The bright hemisphere and the dark one represent the illuminated side and the shadowed side of the isotropic MMs, respectively. (b) Schematic phase transitions of the nonequilibrium assemblies of isotropic photocatalytic TiO_2_@Pt MMs among apolar solids, polar liquids, and polar solids when local interactions are coordinately programmed using UV lights. The inset (region i) shows that Pt nanoparticles are uniformly decorated on the surface of a TiO_2_ microsphere in a TiO_2_@Pt MM.

Considering that isotropic semiconductor micromotors (MMs) may display uniform light-direction-dependent effective orientations irrespective of Brownian rotations [41], we have proposed a design strategy of light-programable nonequilibrium assemblies of isotropic MMs as schematically illustrated in Figure 1. When being illuminated with a beam of light (e.g., vertical upward UV light), an isotropic semiconductor MM will be separated into two hemispheres, including an illuminated one and a shadowed one, because of the limited penetration depth of light in it (left panel in Figure 1(a)). All neighboring isotropic MMs, irrespective of their Brownian rotations, have the same effective orientation, which is defined as the axis joining the poles of the two hemispheres, as indicated by white dashed arrows in Figure 1(a). With the same light-induced effective orientation and surface photochemical reactions, the isotropic MMs generate controllable nonequilibrium chemical gradients, subsequently assuring local phoretic flow fields and specific interaction (Interaction 1 in Figure 1(a)) between neighboring MMs. If the direction of the incident light changes (oblique downward light, middle panel in Figure 1(a)) or an additional light with a different direction (superimposed lights, right panel in Figure 1(a)) is applied, the effective orientation of neighboring isotropic MMs changes accordingly, thereby tuning the nonequilibrium motor-motor interactions (from Interaction 1 to Interaction 2 and 3, Figure 1(a)). Hence, a group of isotropic MMs is expected to be self-organized into collective patterns with light-tunable (positional and orientational) orders.

To implement this design, herein, we select monodispersed TiO_2_ microspheres with uniformly decorated Pt nanoparticles on the surface (TiO_2_@Pt MMs, the inset in Figure 1(b)) as an example in view of their isotropic structure and fuel-free self-propulsion. And UV lights with different directions are used as the stimuli to program local interactions of TiO_2_@Pt MMs by adjusting their global light-induced orientations. Specifically, a vertical upward UV light (UV_Z_, left panel in Figure 1(a)) is applied to introduce local attraction among the TiO_2_@Pt MMs, and an oblique downward UV light (UV_XZ_ or UV_YZ_, middle panel in Figure 1(a)) is applied to set the self-alignment (i.e., phototaxis) and repulsion rules among them. Moreover, the superimposed oblique downward and vertical upward UV lights (e.g., UV_XZ_ and UV_Z_, the right panel in Figure 1(a)) are used to coordinately program the relative strength of local attraction, repulsion, and self-alignment *via* temporal and spatial control of the “on-off” state and intensity (*I*) of the constituent UV lights. With the above light-programmable local interactions, the TiO_2_@Pt MMs are envisioned to reversibly and rapidly transform among three collective formations: apolar solids (i.e., immobile colloidal crystal), polar liquids (i.e., phototactic colloidal stream), and polar solids (i.e., phototactic colloidal crystal) (Figure 1(b)).

**Figure 2:**
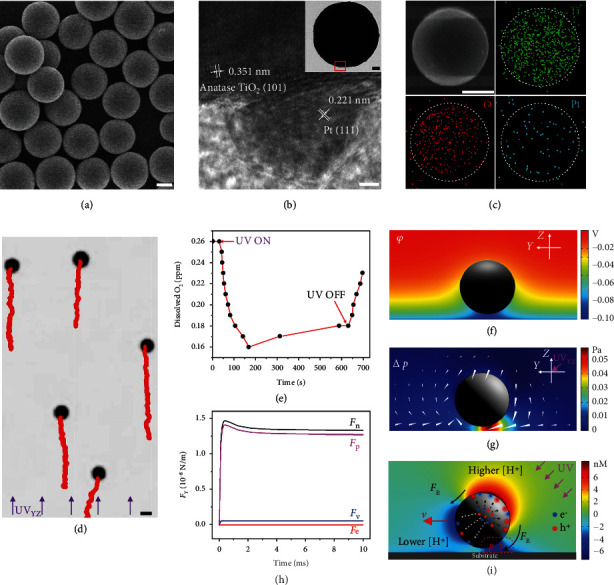
Characterizations and phototaxis of TiO_2_@Pt MMs. SEM (a), TEM (b), and elemental EDX mapping (c) analysis of the TiO_2_@Pt MMs. Scale bars are 1 *μ*m, 2 nm, 1 *μ*m, and 200 nm in (a–c) and the inset in (b), respectively. (d) Negative phototaxis of the dispersed TiO_2_@Pt MMs under an oblique downward UV_YZ_ irradiation with an *I* of 1 W/cm^2^. Red curves represent their trajectories in 3 s. The scale bar is 2 *μ*m. (e) The concentration changes of the dissolved oxygen in an aqueous suspension of TiO_2_@Pt MMs (1 mg/mL) over time when the UV irradiation (*I* = 1 W/cm^2^) is on and off. (f) Numerical simulation of the electric potential (*φ*) of the EDL of a TiO_2_@Pt MM (*ζ*_*p*_ = −25 mV) residing near a glass substrate (*ζ*_*w*_ = −85 mV). (g) Numerical simulations of the increment of electric body force (white triangles) and fluid pressure increment (∆*p*, color background) around a TiO_2_@Pt MM residing near the glass substrate under UV_YZ_ irradiation. (h) The various components of the calculated horizontal driving force (*F*_Y_) acting on the TiO_2_@Pt MM over time obtained from the simulated results in (g), including the electrostatic force (*F*_e_), viscous force (*F*_v_), and fluid-pressure force (*F*_p_) in the *Y* direction (propulsion direction), resulting in a net driving force (*F*_n_). The calculated forces have a unit of N/m since the numerical model is two-dimensional. (i) The propulsion mechanism of a TiO_2_@Pt MM under the oblique downward UV irradiation (purple arrows). The color background depicts the [H^+^] increment (∆[H^+^]) obtained by numerical simulations.

The monodispersed TiO_2_@Pt MMs were synthesized by modifying anatase TiO_2_ microspheres with Pt nanoparticles (see details in Materials and Methods). The obtained TiO_2_@Pt MMs have a spherical morphology with a nearly uniform size of 2 *μ*m (Figure 2(a)), and the size of Pt nanoparticles on their surface is about 5 nm (Figure 2(b)). Energy dispersive X-ray (EDX) mapping of Ti, O, and Pt elements reveals that the TiO_2_@Pt MMs have a nearly isotropic structure (Figure 2(c)). In addition, the TiO_2_@Pt MMs have a negatively charged surface, and their zeta potential (*ζ*_*p*_) was measured to be -25 mV. Under the oblique downward UV_YZ_ irradiation with an *I* of 1 W/cm^2^, the scattered isotropic TiO_2_@Pt MMs, among which interactions were negligible, showed negative phototaxis in water with an average velocity (*v*) of 4.5 *μ*m/s and nearly straight-line trajectories due to their uniform oblique head-down orientations (Figure 2(d) and Movie S1).

It is widely considered that noble metal-modified photocatalysts can induce photocatalytic water splitting to generate H_2_ and O_2_ molecules due to both efficient emergence and separation of photogenerated electron-hole pairs [46–50]. However, after carefully monitoring the concentration change of the dissolved H_2_ and O_2_ in the aqueous suspension of the TiO_2_@Pt MMs (1 mg/mL) under UV irradiation (*I* = 1 W/cm^2^), it was confirmed that no H_2_ could be produced and the dissolved O_2_ was consumed during photocatalysis (Figure 2(e)). Therefore, the reasonable photocatalytic reactions on a TiO_2_@Pt MM are the reduction of O_2_ and oxidation of water, producing O_2_^∙−^ and OH^∙^ [51, 52]. As the photogenerated O_2_^∙−^ and OH^∙^ are highly reactive and further react to produce hydrogen peroxide, the overall reactions can be described as Equations (1) and (2) [52, 53].

(1) $$\begin{array}{c} 2{\text{H}}_{2}\text{O}+2{\text{h}}^{+}⟶{\text{H}}_{2}{\text{O}}_{2}+2{\text{H}}^{+} \end{array}$$

(2) $$\begin{array}{c} 2{\text{H}}^{+}+{\text{O}}_{2}+2{\text{e}}^{-}⟶{\text{H}}_{2}{\text{O}}_{2} \end{array}$$

Under the oblique downward UV irradiation, a Janus structure with different light exposure (illuminated hemisphere and shadowed one) forms because of the limited penetration depth of UV light in the TiO_2_@Pt MM. As the dominant photocatalytic reaction occurred on the illuminated side of the TiO_2_@Pt MM, the photocatalytic products (i.e., H_2_O_2_) would be asymmetrically distributed across the motor. However, the concentration gradient of H_2_O_2_ molecules could not provide enough driving force for the MMs according to the previous calculation [54]. On the other hand, the photogenerated electrons have higher mobility and a longer lifetime than the photogenerated holes in TiO_2_ and TiO_2_@Pt photocatalysts [55], and thus, few holes can arrive at the shadowed side of the TiO_2_@Pt MMs. According to the condition of electrical neutrality, more holes would react on the illuminated side than electrons, while on the other side is on the contrary. Therefore, the overall reaction on the illuminated side is oxidation (Equation (1)) while on the shadowed one performs reduction (Equation (2)). In this condition, H^+^ would be produced on the illuminated side but consumed on the other side (Equations (1) and (2)), generating a local diffusion-electric field (*E*) to drive the TiO_2_@Pt MMs *via* self-electrophoresis [56].

Previous theoretical calculations and numerical simulations of self-electrophoresis often assumed the thin-Debye layer limit, which means the ratio of the thickness of the EDL (i.e., the Debye screening length *κ*^−1^) to the particle radius *r* is close to zero, as described by Equation (3) [44, 45, 57].

(3) $$\begin{array}{c} \lambda \equiv {\left(\kappa r\right)}^{-1}⟶0 \end{array}$$

However, the simulated EDL of a TiO_2_@Pt MM residing near a glass substrate, which was obtained by the Gouy-Chapman-Stern (GCS) model using COMSOL Multiphysics software, reveals a large average *λ* (~0.5), violating the thin-Debye layer assumption (Figure 2(f)). Hence, the phoretic propulsion of the MMs cannot simply be attributed to the surface electrophoretic slip, but a semibulk flow of the charged liquid in the EDL driven by the local electric field body force (*F*_E_).

(4) $$\begin{array}{c} F_{\text{E}}=-\rho_{e}\nabla \varphi ,. \end{array}$$

Here, *φ* is the electrostatic potential and *ρ*_*e*_ is the volumetric charge density. The numerical simulation of the charged liquid flow under the *F*_E_ (white triangles in Figure 2(g)) indicates that the charged liquid around the MM squeezed into the confined space between the MM and the substrate near the light source, resulting in a fluid pressure increment (∆*p*) there (color background) (Figure 2(g)). The ∆*p* was then confirmed to be the main contribution to the net driving force (*F*_n_) of the MM after calculating various forces acting on it in the *Y* direction, including electrostatic force (*F*_e_), viscous force (*F*_v_), and pressure force (*F*_p_), as shown in Figure 2(h). Surprisingly, there is little contribution of *F*_e_ directly to the translation of the MM when the particle is not regarded as a mass point.

According to the above analysis, the propulsion mechanism of the TiO_2_@Pt MMs is proposed and summarized in Figure 2(i). Under oblique downward UV irradiation, water oxidation mainly occurs on the illuminated side of the TiO_2_@Pt MM to produce H^+^, while the reduction of O_2_ dominates on the shadowed side to consume H^+^ (Equations (1) and (2)). Due to the asymmetric distribution of H^+^ across the MM (color background in Figure 2(i)), a local *E* forms and drives the charged liquid in the EDLs of the MM and the glass substrate to flow from the illuminated side to the shadowed side under *F*_E_ (black arrows in Figure 2(i)). Thus, the fluid pressure in the confined space between the MM and the substrate near the incident light increases sharply and drives the MM away from the light source (negative phototaxis). To verify this mechanism, we have investigated the motion of TiO_2_@Pt MMs upon the oblique downward UV irradiation in aqueous media with various ionic species, such as NaCl, cetyltrimethylammonium bromide (CTAB), and sodium dodecyl sulfate (SDS) [58]. As expected, the TiO_2_@Pt MMs showed a serious reduction in *v* in the medium with NaCl or SDS (1 mM in concentration) due to the electric-field screening by the ions and experienced a direction reversal in that with CTAB (1 mM) because of its known ability to reverse the surface charge of oxide surfaces (Figure S1). Furthermore, when a vertical upward UV light (UV_Z_) was applied, the TiO_2_@Pt MMs showed random Brownian superdiffusion (stochastic walk) because of the inevitable structural imperfection [19], and their vertical phototactic motion in the *Z* direction was inhibited by their gravity (Figure S2).

**Figure 3:**
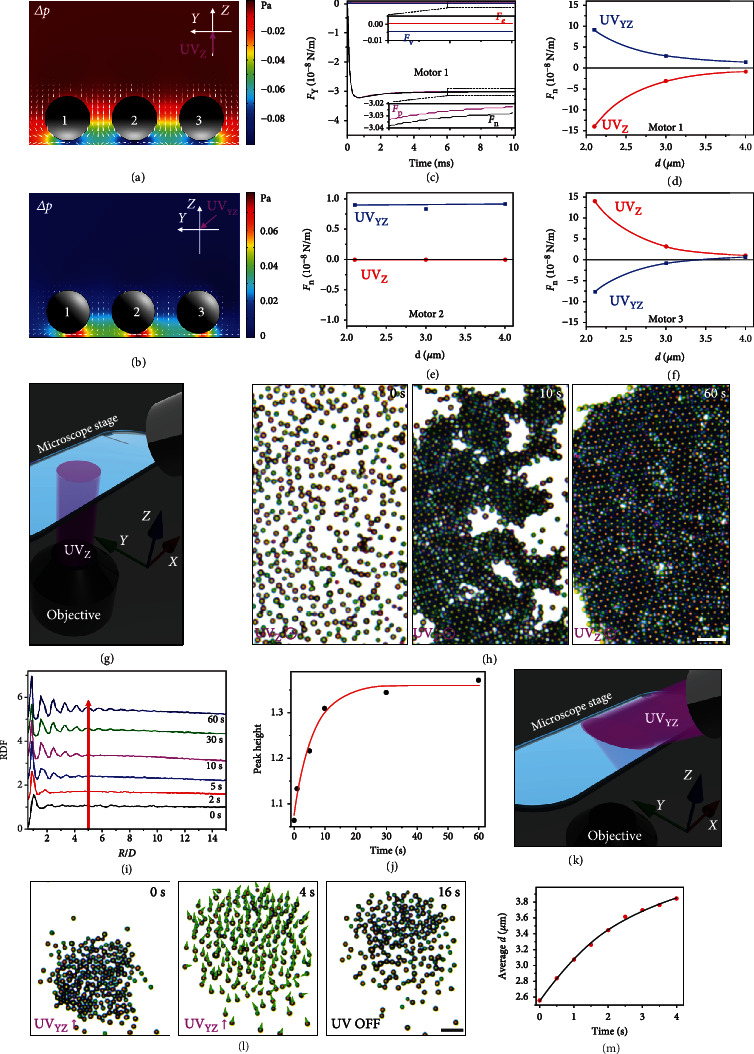
Nonequilibrium assemblies of TiO_2_@Pt MMs under a single UV light. (a, b) Numerical simulations of the electric body force increment (white triangles) and fluid pressure increment (∆*p*, color background) around three TiO_2_@Pt MMs (motors 1, 2, and 3) residing near the glass substrate with a *d* of 3 *μ*m under UV_Z_ (a) and UV_YZ_ irradiation (b). (c) The calculated various horizontal driving force (*F*_Y_) acting on the motor 1 in (a) with the increasing irradiation time under UV_Z_ irradiation. The *F*_n_ acting on motors 1 (d), 2 (e), and 3 (f) with an increasing *d* from 2.1 to 3 and 4 under UV_Z_ (red curves) or UV_YZ_ (blue curves) irradiation, respectively. (g) Schematic illustration of the experimental device of the vertical upward UV_Z_ irradiation. (h) The light-triggered assembly of the TiO_2_@Pt MMs under UV_Z_ irradiation with an *I* of 0.51 W/cm^2^. (i) Radial distribution functions (RDFs) for the TiO_2_@Pt MMs after UV_Z_ exposure for different times as a function of the ratio between the distance from a reference particle (*R*) and the particle diameter (*D*). The curves are vertically shifted for clarity. (j) Height of the peak at *R*/*D* = 5 in the RDF curves in (i) as a function of UV_Z_ irradiation time. (k) Schematic illustration of the experimental device of the oblique downward UV_YZ_ irradiation. (l) The phase transition of an apolar liquid cluster of the TiO_2_@Pt MMs into a polar liquid (i.e., “phototactic stream”) under the UV_YZ_ irradiation with an *I* of 1 W/cm^2^. The scale bars in (h, l) are 10 *μ*m. (m) The variation of *d* between the flocking TiO_2_@Pt MMs over time corresponding to the results in (l).

To probe the distinct orientation-dependent interactions between TiO_2_@Pt MMs under the vertical upward irradiation (UV_Z_) or oblique downward one (UV_XZ_ or UV_YZ_), a three-particle numerical model was established (Figures 3(a) and 3(b)). When UV_Z_ or UV_YZ_ is on, the charged liquid in the EDL (Figure S3) flows from the illuminated side of the TiO_2_@Pt MMs to their shadowed side under the local *E*, generating a pressure decrement (∆*p*, Figure 3(a)) or increment (∆*p*, Figure 3(b)) in the confined spaces between each MM and the substrate, respectively. With the ∆*p*, the intermotor interactions arise, as evidenced by the dominant contribution of *F*_p_ to the received attraction (*F*_n_) of Motor 1 in Figure 3(a) from Motor 2 (Figure 3(c)). Thus, under the UV_YZ_ irradiation, TiO_2_@Pt MMs tend to repel each other owing to the increasing inter-motor pressure, while they attract each other because of the decrement of the intermotor fluid pressure upon UV_Z_ irradiation. When the intermotor distance (*d*) decreases from 4 to 2.1 *μ*m, the net interaction force (*F*_n_) acting on the middle TiO_2_@Pt MM (motor 2) shows negligible changes, and the strength of *F*_n_ (|*F*_*n*_|) on motors 1 and 3 increases sharply (Figures 3(d)–3(f)). The above results suggest that the interactions between TiO_2_@Pt MMs can be reversed from repulsion to attraction by simply changing the direction of incident light from oblique downward to vertical upward, and their strength shows a strong dependence on *d*.

Utilizing the light-direction-switchable orientation-dependent interactions of the TiO_2_@Pt MMs, we are able to control their self-assembly and collective orders. At first, the dispersed TiO_2_@Pt MMs were collected in the observing area of the optical microscope by employing the near-infrared (NIR) light-induced convections [59]. After collection, the local number density of TiO_2_@Pt MMs increased, and an incompact liquid-form cluster appeared near the substrate, in which the MMs were in proximity but without any ordered organization (0 s in Figure S4). When UV_Z_ was applied (Figure 3(g)), as predicted by numerical simulation (Figure 3(a)), the attraction among TiO_2_@Pt MMs arose, and they self-organized into an apolar solid with a long-range ordered lattice structure (i.e., colloidal crystals) (0–60 s in Figure 3(h) and Movie S2). To characterize the kinetics of disorder-to-order transitions of the assemblies of TiO_2_@Pt MMs, a radial distribution function (RDF) was computed from their time-lapse microscopic images (Figure 3(h)) [60, 61]. With the prolonged irradiation time (*t*), peaks in the RDF curves start to emerge (Figure 3(i)) in the first 5 s, and the peak number and height increase gradually. Well-defined peaks up to *R*/*D* = 8 appearing in the RDF curves at a *t* of 60 s (Figure 3(i)) reveal the long-range hexagonal packing of TiO_2_@Pt MMs. The increasing height of the peak at *R*/*D* = 5 as a function of *t* (Figure 3(j)) suggests that the crystallization was achieved at about 30 s. The close observation of the crystallization of a small cluster of TiO_2_@Pt MMs (Movie S3) unravels that they at first self-assembled into small crystallites in regions with a high local number density, as verified by the velocity vectors of the TiO_2_@Pt MMs (0-0.2 s in Figure S4). This is because the TiO_2_@Pt MMs in high-number-density regions had a shorter intermotor *d* and thus experienced stronger mutual attraction than those in low-number-density regions. In addition, the formed small crystallites merged into a large 2D colloidal crystal which was then annealed over time through edge contraction and “surface energy reduction” (7-670 s in Figure S4). When UV_Z_ ceased, the colloidal crystal melted into a liquid-form cluster even after a long term of UV_Z_ irradiation (670 s), exhibiting excellent reversibility (670-700 s in Figure S4). When a small number of passive polystyrene (PS) microspheres (2 *μ*m in size) were added into the system, either negatively charged (*ζ*_*p*_ = −23 mV) or positively charged (*ζ*_*p*_ = +9.4 mV) PS microspheres could be captured by the TiO_2_@Pt MMs and organized into the lattices of the colloidal crystal in the presence of UV_Z_ light (Figure S5). This suggests that the phoretic flow effect, rather than the electrostatic interaction, is responsible for the self-organization of the TiO_2_@Pt MMs.

When irradiated by the UV_YZ_, of which the experimental installation is shown in Figure 3(k), repulsive interaction and self-alignment (collective phototaxis) are introduced among the TiO_2_@Pt MMs. The velocity vectors of individual TiO_2_@Pt MMs (green triangles in Figure 3(l)) indicate that almost all of them moved in the *Y* direction with a high orientational order. In the meantime, during the phototactic flocking, the TiO_2_@Pt MMs repelled each other and the average *d* increased accordingly (Figure 3(m)), consistent with the numerical simulation results in Figure 3(b). These results suggest that the apolar liquid-form cluster of TiO_2_@Pt MMs transformed into a polar liquid (i.e., “phototactic stream”) (Movie S4). Once UV_YZ_ was removed, the “phototactic stream” stopped, and the TiO_2_@Pt MMs then gradually reunited into a liquid-form cluster again within 12 s (4–16 s in Figure 3(l)) due to the diffusiophoretic attraction [53].

**Figure 4:**
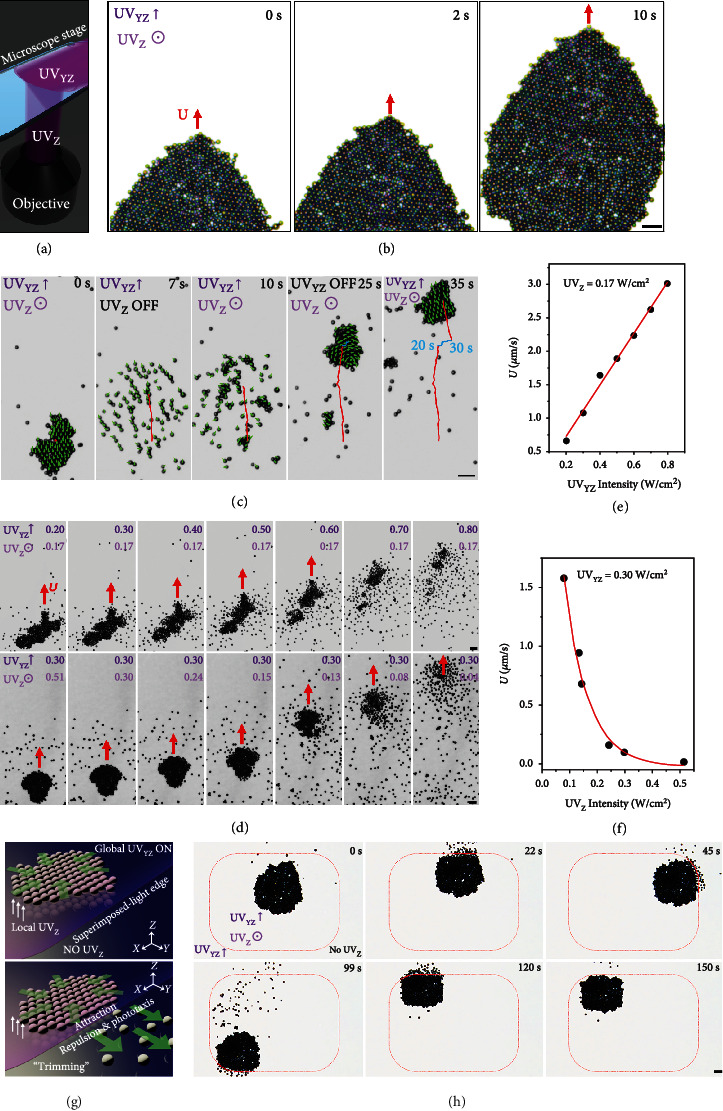
Nonequilibrium assemblies of TiO_2_@Pt MMs under the superimposed UV irradiation. (a) Schematic illustration of the experimental device of the superimposed UV_YZ_ and UV_Z_ irradiation. (b) Time-lapse microscopic images depicting the negative phototaxis of a “phototactic colloidal crystal” of the TiO_2_@Pt MMs under the superimposed UV_YZ_ (*I* = 1 W/cm^2^) and UV_Z_ (*I* = 0.3 W/cm^2^) irradiation. (c) Dynamic phase transitions of a “phototactic colloidal crystal” when UV_YZ_ (*I* = 1 W/cm^2^) and UV_Z_ (*I* = 0.51 W/cm^2^) are alternatively turned on and off. Green triangles are velocity vectors of individual TiO_2_@Pt MMs. Red curves are phototactic trajectories of the whole group when UV_YZ_ is on, and blue curves are its trajectories from 20 to 30 s when UV_YZ_ is off. On-the-fly phase transitions (d) and flocking velocity (*U*) (e, f) of the “phototactic colloidal crystals” under the superimposed irradiation as a function of *I* of UV_YZ_ (e) and UV_Z_ (f) when keeping UV_Z_ at 0.17 W/cm^2^ and UV_YZ_ at 0.30 W/cm^2^, respectively. Schematic illustration (g) and time-lapse microscopic images (h) depicting the “trimming” of an irregular colloidal crystal into a rectangular one under the superimposed irradiation consisting of a global UV_YZ_ (*I* = 1 W/cm^2^) and a local UV_Z_ (*I* = 0.3 W/cm^2^). Scale bars are 10 *μ*m.

Due to the light-direction-switchable interactions from attraction to self-alignment/repulsion between TiO_2_@Pt MMs, it is natural for us to wonder what will happen when the oblique downward (UV_XZ_ or UV_YZ_) and vertical upward (UV_Z_) lights are applied simultaneously (the superimposed UV irradiation, Figure 4(a)). Figure 4(b) gives a typical experimental example, in which the assembled colloidal crystal of TiO_2_@Pt MMs under UV_Z_ irradiation (*I* = 0.3 W/cm^2^) performed negative phototaxis in the *X*‐*Y* plane with a stable ordered lattice structure when UV_YZ_ (*I* = 1 W/cm^2^) was turned on (Movie S5). This result reveals that a polar solid (i.e., “phototactic colloidal crystal”) has been realized under the superimposed irradiation (Figure 4(b)), and the constituent TiO_2_@Pt MMs can hold their positions in the crystal during phototaxis. To investigate the dynamic crystallization and phase transitions of the “phototactic colloidal crystal,” the dynamic phase transitions of a small “phototactic colloidal crystal” were recorded when UV_YZ_ and UV_Z_ were alternatively turned on and off (Figure 4(c) and Movie S6). Specifically, the “phototactic colloidal crystal” formed under the superimposed irradiation immediately melted into a “phototactic stream” because the attraction among the TiO_2_@Pt MMs disappeared when the UV_Z_ was switched off (0-7 s in Figure 4(c)). Once the UV_Z_ was turned on again (10 s in Figure 4(c)), the TiO_2_@Pt MMs self-organized into small crystallites again when flocking in negative phototaxis and gradually merged into a large “phototactic colloidal crystal”. Then, the “phototactic colloidal crystal” stopped when UV_YZ_ was off (20-30 s in Figure 4(c)). The slight drift (blue trajectory from 20 to 30 s in Figure 4(c)) of the colloidal crystal was resulted from the attraction of another large colloidal crystal outside the screen. Once applying UV_YZ_ again, the colloidal crystal could be reactivated and perform phototaxis (30-35 s in Figure 4(c)).

The phase transitions of the “phototactic colloidal crystal” under the superimposed irradiation can also be controlled on the fly by adjusting the *I* of UV_YZ_ or UV_Z_ (Movie S7). When the *I* of UV_YZ_ was gradually increased from 0.2 to 0.8 W/cm^2^ but that of UV_Z_ was kept at 0.17 W/cm^2^, the “phototactic colloidal crystal” melted into a “phototactic stream” on the fly because the repulsion gradually dominated over the attraction among the TiO_2_@Pt MMs (top row in Figure 4(d)). In addition, the increasing *I* of UV_YZ_ also enhanced the phototactic velocity (*U*) of the flocking TiO_2_@Pt MMs either in the form of “phototactic colloidal crystal” or “phototactic stream” (Figure 4(e)). Similarly, when the *I* of UV_Z_ was gradually decreased from 0.51 to 0.04 W/cm^2^ with a constant UV_YZ_ of 0.3 W/cm^2^, the “phototactic colloidal crystal” also melted on the fly because of the decreasing intermotor attraction (bottom row in Figure 4(d)). It is noted that UV_Z_ with a high *I* often leads to deceleration of the “phototactic colloidal crystal” (Figure 4(f)), which is probably induced by the decreased asymmetry of the TiO_2_@Pt MMs (right panel in Figure 1(a)). The *I*-dependent on-the-fly phase transitions of the “phototactic colloidal crystal” can be rationalized by the fact that the change in *I* results in the change of photon flux and thus the photocatalytic reaction rate and local *E* [62], thereby modulating the relative strength of phoretic attraction and repulsion among TiO_2_@Pt MMs. Concluded from Figures 4(d)–4(f), whether we strengthen intermotor repulsion by elevating the *I* of UV_YZ_ or weaken attraction by attenuating the *I* of UV_Z_, the “phototactic colloidal crystal” melts from the surface once the intermotor repulsion dominates over attraction. This is because the peripheral MMs have larger local free volumes than those in the bulk and therefore have a greater propensity to melt. After the surface melting, the “phototactic colloidal crystals” can further fracture into small crystallites and finally disassemble into dispersed motors (Figure 4(d) and Movie S7). The above results suggest that the superimposed UV lights (UV_YZ_ and UV_Z_) can be used to program local attraction, repulsion, and self-alignment among TiO_2_@Pt MMs *via* temporal control of the “on-off” state and *I* of the constituent UV lights. Thus, the colloidal crystal can be maneuvered to perform nonequilibrium phase transitions on the fly from apolar solids and polar liquids to polar solids.

Engineering colloidal crystals with predesigned patterns is highly desired especially for constructing functional microdevices, such as photonic crystals and microlens arrays [63–66]. However, the reported colloidal crystals usually have an irregular shape because of the difficulties in the temporal and spatial control of the “bottom-up” assembly process [27]. By virtue of the light-direction-dependent interactions of TiO_2_@Pt MMs and the high spatial resolution of light, here, we propose a “top-down” light-trimming strategy to engineer the colloidal crystals. To demonstrate this strategy, we at first built superimposed irradiation consisting of a global UV_YZ_ (*I* = 1 W/cm^2^) and a local rectangular UV_Z_ (*I* = 0.3 W/cm^2^) (top panel in Figure 4(g)). As the colloidal crystal retains its ordered configuration in the superimposed irradiation area and disassembles into dispersed phototactic motors out it (bottom panel in Figure 4(g)), the edges of the superimposed irradiation area (i.e., the edges of the rectangular UV_Z_ spot) can act as sharp “knifes” to trim the colloidal crystal (Movie S8). As demonstrated in Figure 4(h), by moving the microscope stage toward its topside, the undesired part of an irregular colloidal crystal was moved out of the superimposed irradiation area, and thus, it was “cut” off from the crystal when melting into a “phototactic stream.” Through repeating the “cutting” process several times at different superimposed-light edges (45-120 s in Figure 4(h)), the irregular colloidal crystal was finally trimmed into a rectangular one (150 s in Figure 4(h)). If the local rectangular UV_Z_ in the superimposed irradiation was replaced by a circular one, a quadrilateral swarm can be cut into a circular one (Figure S6 and Movie S9). It is rational to anticipate that “colloidal crystals” with more sophisticated patterns could be engineered with more delicate superimposed-light “knifes” (e.g., the customized apertures with different shapes and sizes) and more advanced “carving apparatuses” (e.g., programmable automated microscope stages).

## 3. Discussion

In summary, we have demonstrated that isotropic TiO_2_@Pt MMs in water can be organized into nonequilibrium assemblies with light-programmable phase transitions (e.g., from apolar solids and polar liquids to polar solids) utilizing their uniform light-direction-adaptive effective orientations and local interactions irrespective of their Brownian rotations. Numerical simulations reveal that the fluid pressure induced by the persistent semibulk phoretic flow from the illuminated to the shadowed side determines the propulsion and the interactions of TiO_2_@Pt MMs. Upon the vertical upward UV_Z_ irradiation, isotropic TiO_2_@Pt MMs can freeze into apolar solids (i.e., colloidal crystals) due to the light-induced local attraction, while they transform into polar liquids (i.e., “phototactic streams”) because of their dilatational negative phototaxis under the local repulsion and self-alignment set by the oblique downward (UV_XZ_ or UV_YZ_) irradiation. By applying superimposed irradiation consisting of vertical upward (UV_Z_) and oblique downward (UV_XZ_ or UV_YZ_) lights, isotropic TiO_2_@Pt MMs self-organize into polar solids (i.e., “phototactic colloidal crystals”). By adjusting the “on-off” state and *I* of the constituent UV lights, the relative strength of the local intermotor interactions can be modulated, and thus, the phase transitions and phototactic velocity of the “phototactic colloidal crystals” can be reversibly controlled on the fly. Furthermore, based on the orientation-dependent interactions among constituent isotropic motors, a “top-down” light-trimming strategy is proposed to “cut” the colloidal crystal into the desired pattern using the local superimposed-light edges as “knifes.” Such versatile nonequilibrium assemblies can be employed as an excellent model system for the study of dynamic processes of phase transitions. The light-steering strategy for isotropic motors (or spherical semiconductor particles) proposed here may offer a facile technology for on-site building intelligent functional microdevices, such as reconfigurable swarming micro/nanorobots, motile responsive photonic crystals, dynamic motile microlens arrays, and adaptive motile plasmonic devices.

## 4. Materials and Methods

### 4.1. Fabrication of TiO_2_@Pt MMs

Monodisperse anatase TiO_2_ microspheres with a size of 2 *μ*m were first prepared according to the previous report [67]. Briefly, 0.35 mL formic acid was added into 30 mL ethanol, followed by the addition of 0.425 mL of titanium(IV) isopropoxide to form a solution, which was stirred for 10 min, then transferred into a 50 mL Teflon-lined stainless steel autoclave and kept at 150°C for 1 h. After the supernatant was discarded, the white precipitate was dispersed in newly added ethanol by ultrasonic dispersion, then collected by centrifugation, and washed with ethanol and water three times. The product was dried under 60°C for 12 h and then calcined at 400°C for 2 h.

To fabricate TiO_2_@Pt MMs, 10 mg monodisperse anatase TiO_2_ microspheres were dispersed in 2.3 mL water, followed by the addition of 115 *μ*L chloroplatinic acid hexahydrate aqueous solution (0.01 M) into the mixture in ultrasonic dispersion for 1 h. 170 *μ*L sodium borohydride aqueous solution (0.01 M) was added before the mixture was in ultrasonic dispersion for another 1 h. The product was collected by centrifugation and washed with water several times.

### 4.2. Characterization

Scanning electron microscopy (SEM) images and energy-dispersive X-ray (EDX) element mapping analysis were obtained using a JEM-7500F field-emission SEM (Japan). The transmission electron microscopy (TEM) images were captured by JEM-F200 (Japan). The zeta potential of all particles was measured by NanoBrook 90 Plus Zeta (US). The UV light intensity was measured by a light intensity meter (PM100D, US).

### 4.3. Propulsion and Self-Organization of TiO_2_@Pt MMs

A 50 *μ*L aqueous suspension of the TiO_2_@Pt MMs (1 mg/mL) was dropped onto a glass slide previously cleaned alternately with water and ethanol several times. A custom experimental setup was built to control the propulsion and self-organization of the MMs by light. A UV lamp with a wavelength of 365 nm and a maximum *I* of 1 W/cm^2^ (SZ Lamplic Technology) was used as the oblique downward light source (UV_XZ_ or UV_YZ_), which was set above the substrate with an incident angle of 45^o^. The UV light from the built-in Leica EL6000 light source of an inverted optical microscope (Leica DMI 3000B) was used as the vertical upward light source (UV_Z_). To observe the light-controlled propulsion of single TiO_2_@Pt MMs, the oblique downward (UV_XZ_ or UV_YZ_) light source was used to power the MMs. To investigate the assembly and flocking behavior of the TiO_2_@Pt MMs, they at first were collected at the center of the observation area by the near-infrared (NIR) light-induced convections using a NIR laser lamp with a maximum *I* of 2 W/cm^2^ [59]. Then, the crystallization, phase transitions, and phototactic flocking of the TiO_2_@Pt MMs were investigated by controlling the on/off state and *I* of UV lights. All videos were analyzed by Video Spot Tracker V08.01 software. The instantaneous velocity vectors of the particles were analyzed by commercial PIV software (PIV View 2C) [68]. To calculate the radial distribution function (RDF), which describes the number of particles positioned at a (center-to-center) distance *R* away from a given reference particle, quantitative analysis of the high-magnification images (typically containing about 2000 particles) was carried out using the “radial distribution function” macro of ImageJ software [60].

### 4.4. Numerical Simulation

The governing equations of the numerical simulations are given in Supplementary Materials. The simulations were performed by using the diffusions, electrostatics and creeping flow modules of COMSOL Multiphysics software [57]. In the simulation, a two-dimensional (2D) model was built up by placing a TiO_2_@Pt MM in the middle of a cuboid cell (120 × 100*μ*m) filled with water with a glass substrate. To virtualize the electric double layer (EDL) of a motor residing near a glass substrate in water, the surface charge density of the substrate (*ρ*_*w*_) and that of the motor (*ρ*_*p*_) were first calculated using a one-dimensional (1D) steady-state model based on their zeta potentials according to Gouy-Chapman-Stern (GCS) theory, in which the thickness of the Stern layer (*x*_*S*_) was set to be 0.5 nm [69]. Then, the calculated *ρ*_*w*_ and *ρ*_*p*_ were set as the boundary condition in the COMSOL model, and the EDL was virtualized by simulating the *φ* and the distribution of H^+^ and OH^−^ ions using the diffusions and electrostatics modules at a steady state. To analyze the *F*_n_ of the MM, the local electric field (*E*) due to the asymmetric generation and consumption of H^+^ and the fluid flows driven by the local *E* were simulated. The release and consumption rate of H^+^ on the illuminated and shadowed surface of the TiO_2_@Pt MM was set to be 1 × 10^−7^ mol/(m^2^·s), respectively. The diffusion coefficient of H^+^ and OH^−^ was set to be 9.31 × 10^−9^ and 5.27 × 10^−9^ m^2^/s, respectively. The zeta potential *ζ*_*p*_ of the TiO_2_@Pt MM was set to be -25 mV according to the result of zeta potential measurement, and that of the glass substrate (*ζ*_*w*_) was set to be -85 mV [70]. The calculated *F*_n_ was obtained with a unit of N/m by integrating the total stress (N/m^2^ in unit) at each point over the circumference of the two-dimensional motor model. To simulate the light-direction-dependent interactions between the TiO_2_@Pt MMs, the local *E* and fluid flows were also simulated by placing three TiO_2_@Pt MMs with different *d* in the above model.

## Data Availability

All data are available in the main text or the supplementary materials.

## Supplementary Materials

Supplementary Materials Fig. S1: the normalized *v* of the TiO_2_@Pt MMs in the water and other aqueous media with different ionic species (1 mM), including NaCl, CTAB, and SDS, under UV_YZ_ irradiation. Fig. S2: trajectories of the TiO_2_@Pt MMs under UV_Z_ irradiation. Fig. S3: numerical simulation of the electric potential (*φ*) of the EDL of three TiO_2_@Pt MMs (*ζ*_*p*_ = −25 mV) residing near a glass substrate (*ζ*_*w*_ = −85 mV). Fig. S4: time-lapse microscopic images depicting the assembly, annealing, and disassembly of TiO_2_@Pt MMs in response to UV_Z_ irradiation. Fig. S5: assembly of negatively charged (PS(-)) and positively charged PS microspheres (PS(+)) by the TiO_2_@Pt MMs under UV_Z_ irradiation. Fig. S6: time-lapse microscopic images depicting the “trimming” of a rectangular colloidal crystal into a circular one under the superimposed irradiation consisting of a global UV_XZ_ (*I* = 1 W/cm^2^) and a local circular UV_Z_ (*I* = 0.3 W/cm^2^). Movie S1: phototaxis of single TiO_2_@Pt MMs under UV_YZ_ irradiation. Movie S2: crystallization of swarming TiO_2_@Pt MMs under UV_Z_ irradiation. Movie S3: crystallization of a small cluster of TiO_2_@Pt MMs under UV_Z_ irradiation. Movie S4: dilatational phototaxis of swarming TiO_2_@Pt MMs under UV_YZ_ irradiation. Movie S5: phototaxis of a colloidal crystal of TiO_2_@Pt MMs under the superimposed UV_YZ_ and UV_Z_ irradiation. Movie S6: dynamic phase transitions of a “phototactic colloidal crystal” by alternatively turning the constituent UV lights of the superimposed irradiation on and off. Movie S7: on-the-fly phase transitions of the “phototactic colloidal crystals” by adjusting the light intensity of the superimposed UV lights. Movie S8: “trimming” an irregular colloidal crystal into a rectangular pattern under the superimposed irradiation consisting of a global UV_YZ_ and a local rectangular UV_Z_. Movie S9: “trimming” a large rectangular colloidal crystal into a circular one under the superimposed irradiation consisting of a global UV_XZ_ and a local circular UV_Z_.
