# Supplementary material for: Light-Programmable Assemblies of Isotropic Micromotors
Source: Research (Wash D C). 2022 Jul 6;2022:9816562. doi: 10.34133/2022/9816562 (PMC9297725; doi:10.34133/2022/9816562)
Supplement: Supplementary Materials — Fig. S1: the normalized v of the TiO2@Pt MMs in the water and other aqueous media with different ionic species (1 mM), including NaCl, CTAB, and SDS, under UVYZ irradiation. Fig. S2: trajectories of the TiO2@Pt MMs under UVZ irradiation. Fig. S3: numerical simulation of the electric potential (φ) of the EDL of three TiO2@Pt MMs (ζp = −25 mV) residing near a glass substrate (ζw = −85 mV). Fig. S4: time-lapse microscopic images depicting the assembly, annealing, and disassembly of TiO2@Pt MMs in response to UVZ irradiation. Fig. S5: assembly of negatively charged (PS(-)) and positively charged PS microspheres (PS(+)) by the TiO2@Pt MMs under UVZ irradiation. Fig. S6: time-lapse microscopic images depicting the “trimming” of a rectangular colloidal crystal into a circular one under the superimposed irradiation consisting of a global UVXZ (I = 1 W/cm2) and a local circular UVZ (I = 0.3 W/cm2). Movie S1: phototaxis of single TiO2@Pt MMs under UVYZ irradiation. Movie S2: crystallization of swarming TiO2@Pt MMs under UVZ irradiation. Movie S3: crystallization of a small cluster of TiO2@Pt MMs under UVZ irradiation. Movie S4: dilatational phototaxis of swarming TiO2@Pt MMs under UVYZ irradiation. Movie S5: phototaxis of a colloidal crystal of TiO2@Pt MMs under the superimposed UVYZ and UVZ irradiation. Movie S6: dynamic phase transitions of a “phototactic colloidal crystal” by alternatively turning the constituent UV lights of the superimposed irradiation on and off. Movie S7: on-the-fly phase transitions of the “phototactic colloidal crystals” by adjusting the light intensity of the superimposed UV lights. Movie S8: “trimming” an irregular colloidal crystal into a rectangular pattern under the superimposed irradiation consisting of a global UVYZ and a local rectangular UVZ. Movie S9: “trimming” a large rectangular colloidal crystal into a circular one under the superimposed irradiation consisting of a global UVXZ and a local circular UVZ. [file 9816562.f1.zip › Che-Supplementary Materials.docx]

Supplementary Materials for

**Light-Programmable Assemblies of Isotropic Micromotors**

Shengping Che^1^†, Jianhua Zhang^1,2^†, Fangzhi Mou^1^*, Xia Guo^1^, Joshua E. Kauffman^2^, Ayusman Sen^2^, Jianguo Guan^1^*

*Corresponding authors. Email: [moufz@whut.edu.cn](mailto:moufz@whut.edu.cn) (F. Mou), [guanjg@whut.edu.cn](mailto:guanjg@whut.edu.cn) (J. Guan)

†These authors contributed equally to this work.

Supplementary Text

**Governing equations for numerical simulation.**

The compact electric double layer (EDL) is considered as a region with uniform dielectrics and constant electric field, and thus, according to the Gauss' law, the surface charge density of the substrate ($\rho_{w}$) and that of the motor ($\rho_{p}$) are given by,

$$\rho_{w}=\frac{\varepsilon_{0}\varepsilon_{r}(\varphi_{w}-\zeta_{w})}{x_{S}}$$

$$\rho_{p}=\frac{\varepsilon_{0}\varepsilon_{r}(\varphi_{p}-\zeta_{p})}{r^{2}(\frac{1}{r}-\frac{1}{r+x_{S}})}$$

Here, $\varphi_{w}$ and $\varphi_{p}$ are the potential at the substrate surface and the particle surface, while $\zeta_{w}$ and $\zeta_{p}$ are the zeta potential of the substrate and the motor, respectively. The $r$ is the radius of the spherical particle and $x_{S}$ is the thickness of the Stern layer. $\varepsilon_{0}$ is the vacuum permittivity, and $\varepsilon_{r}$ is the relative permittivity of the fluid medium.

The distribution of various ions originates from the fluxes of the ions from the surface of the particles, and is further affected by the diffusion, convection and migration of ions, which is given by the Nernst-Planck equation modified by the convection-diffusion equation,

$$J_{i}=uc_{i}-D_{i}\nabla c_{i}-\frac{z_{i}FD_{i}c_{i}\nabla\varphi}{RT}$$

where $u$ is the fluid velocity, $F$ is the Faraday constant, $\varphi$ is the electrostatic potential, *R* is the gas constant, *T* is the absolute temperature, and$c_{i}$, $D_{i}$, $z_{i}$ are the concentration, diffusion coefficient, and charge of species *i*, respectively.

The electric potential ($\varphi$) is calculated using the Poisson equation,

$$-\varepsilon_{0}\varepsilon_{r}\nabla^{2}\varphi=\rho_{e}=F\left( z_{+}c_{+}+z_{-}c_{-} \right)$$

where $\rho_{e}$ is the volumetric charge density, $z_{+}$ and $z_{-}$ are the charges of the cations and the anions, $c_{+}$ and $c_{-}$ are the concentrations of the cations and the anions, respectively. The zero potential is inside the solution at infinity, the same below.

The flow field is governed by the Navier-Stokes equations,

$$\rho\frac{du}{dt}=-\nabla p+\rho F_{E}+\eta\nabla^{2}u$$

where $\rho$ is the fluid density, $p$ is the pressure, $\eta$ is the fluid viscosity and $F_{E}$ is the body force resulting from the electric field force on a charged liquid,

$$F_{E}=-\rho_{e}\nabla\varphi$$

According to the model, the driving force (*F*_n_) the motor consists of two main forces, namely, electrostatic force and fluid force, and *F*_n_ at any point on the motor’s surface is,

$$F_{n}=\rho_{p}\nabla\varphi+F_{stress}$$

The fluid force $F_{stress}$ can be further divided into the pressure component (*F*_p_) and the viscous component (*F*_k_). The total *F*_n_ acting on a motor is calculated by integrating the electrostatic and fluid force on the surface of the MM in the direction parallel to the substrate surface.

Supplementary Figures


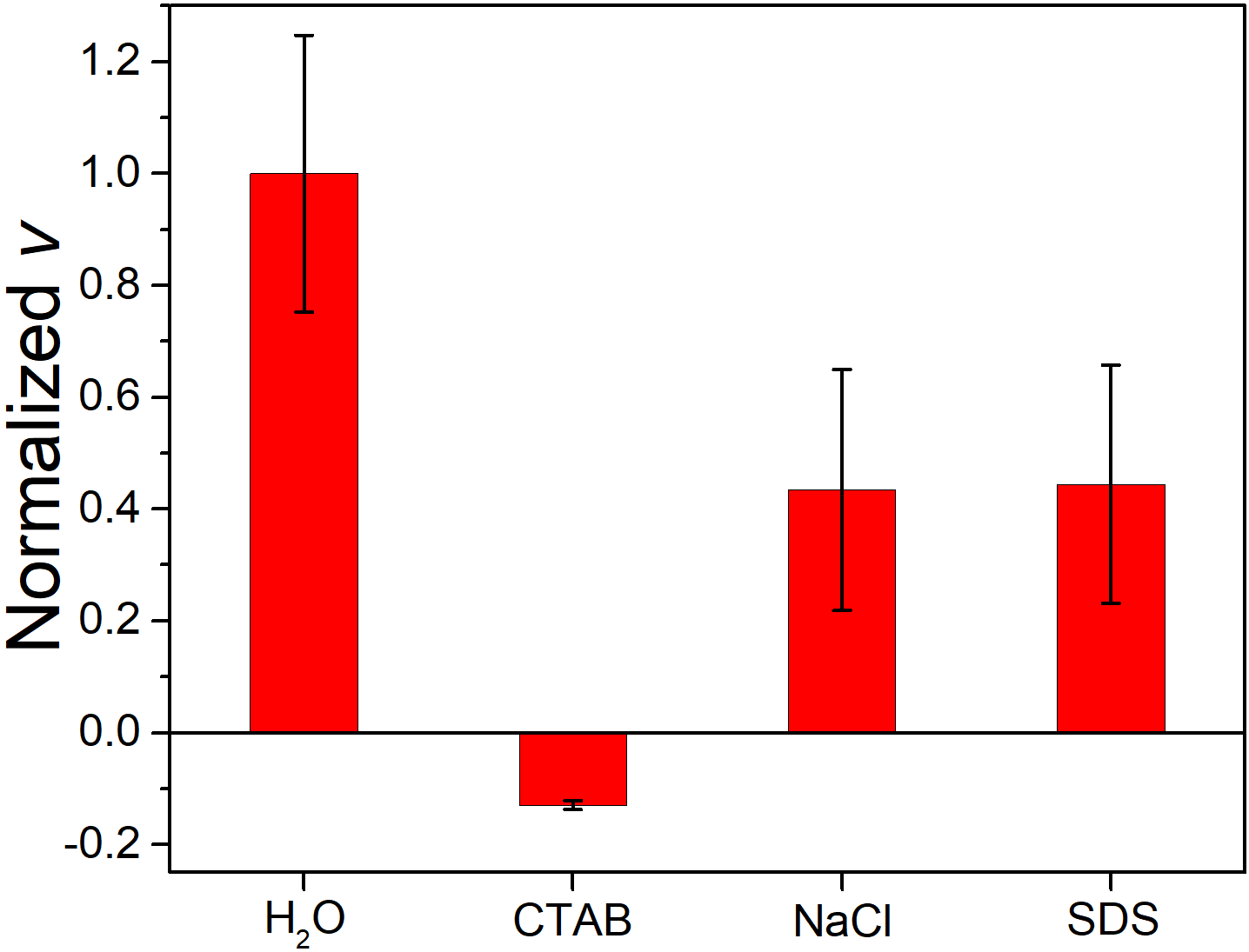


Fig. S1. The normalized *v* of the TiO_2_@Pt MMs in the water and other aqueous media with different ionic species (1 mM), including NaCl, CTAB, and SDS, under UV_YZ_ irradiation.


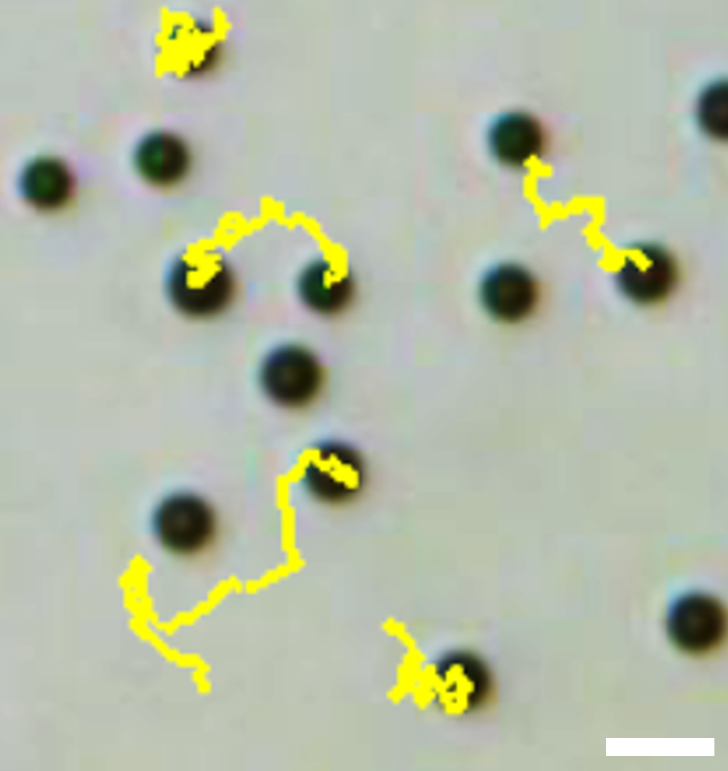


Fig. S2. Trajectories of the TiO_2_@Pt MMs under UV_Z_ irradiation. Scale bar, 5 μm.


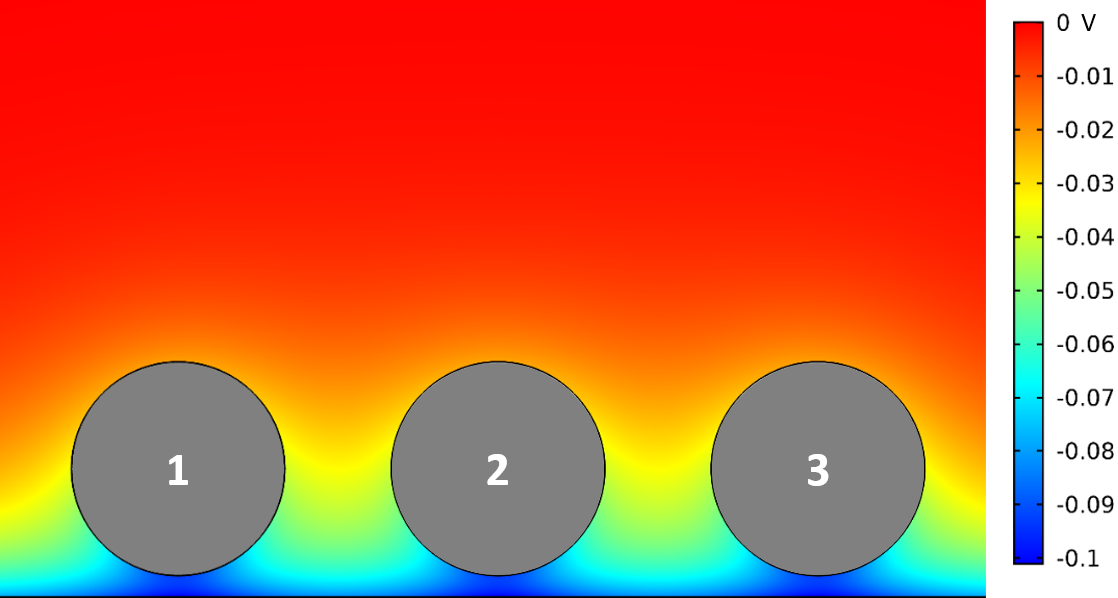


**Fig. S3.** Numerical simulation of the electric potential (*φ*) of the EDL of three TiO_2_@Pt MMs ($\zeta_{p}$ = -25 mV) residing near a glass substrate ($\zeta_{w}$ = -85 mV).


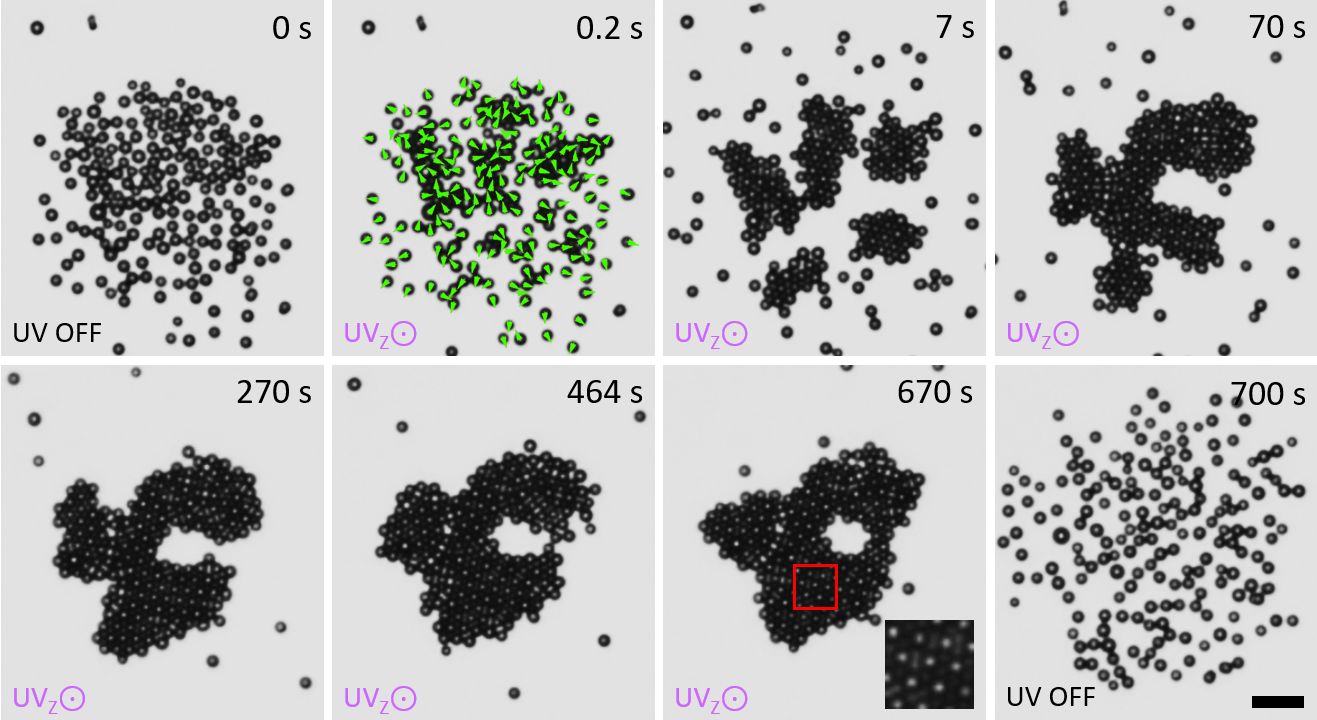


Fig. S4. Time-lapse microscopic images depicting the assembly, annealing and disassembly of TiO_2_@Pt MMs in response to UV_Z_ irradiation. Scale bar, 10 μm.


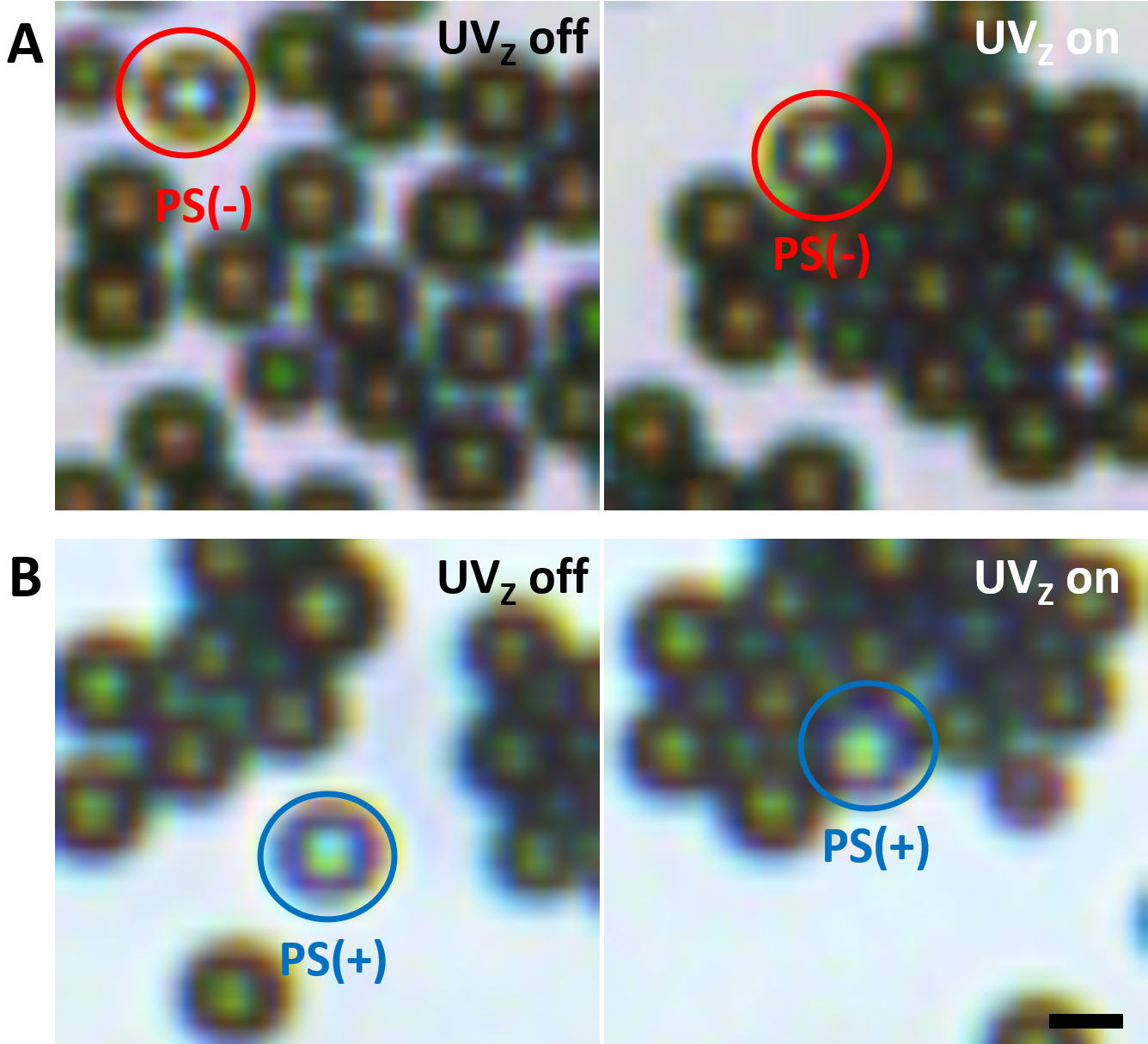


Fig. S5. Assembly of (A) negatively-charged (PS(-)) and (B) positively-charged PS microspheres (PS(+)) by the TiO_2_@Pt MMs under UV_Z_ irradiation, respectively. Scale bar, 10 μm.


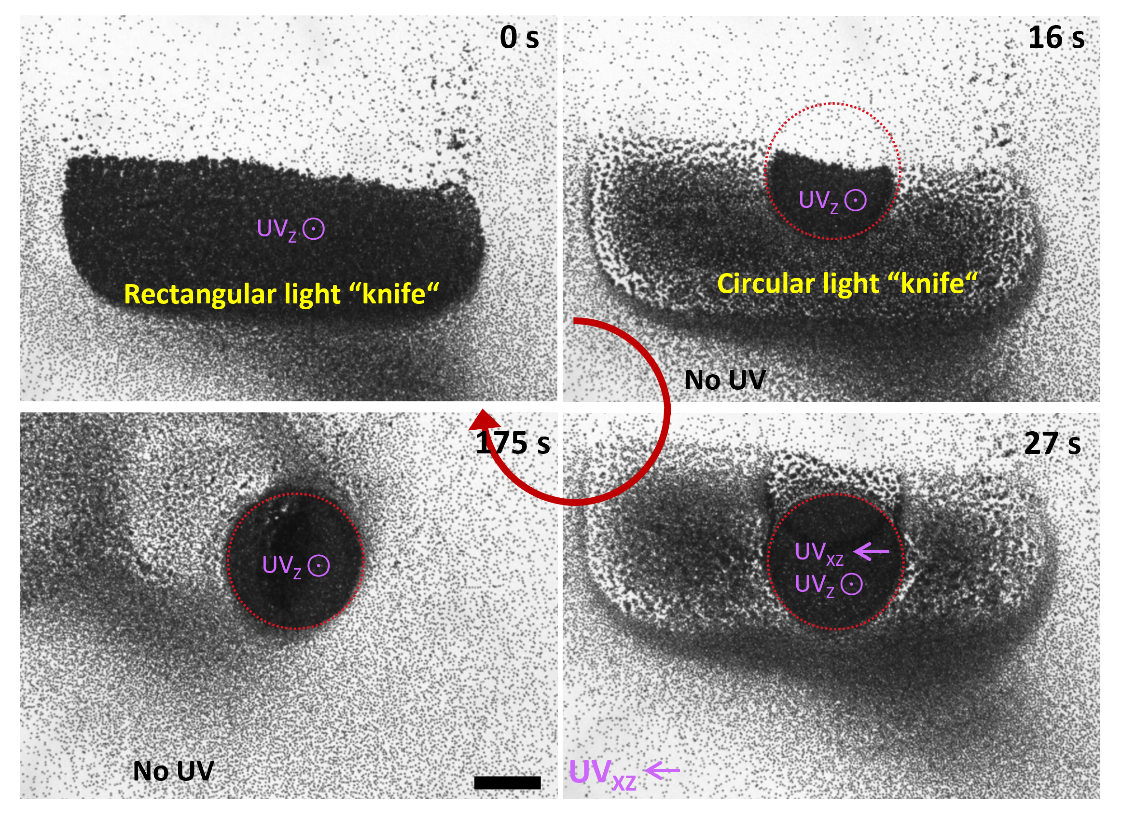


Fig. S6. Time-lapse microscopic images depicting the “trimming” of a rectangular colloidal crystal into a circular one under the superimposed irradiation consisting of a global UV_XZ_ (*I* = 1 W/cm^2^) and a local circular UV_Z_ (*I* = 0.3 W/cm^2^). Scale bars are 100 μm.

Supplementary Movies

Movie S1.

Phototaxis of single TiO_2_@Pt MMs under UV_YZ_ irradiation.

Movie S2.

Crystallization of swarming TiO_2_@Pt MMs under UV_Z_ irradiation.

Movie S3.

Crystallization of a small cluster of TiO_2_@Pt MMs under UV_Z_ irradiation.

Movie S4.

Dilatational phototaxis of swarming TiO_2_@Pt MMs under UV_YZ_ irradiation.

Movie S5.

Phototaxis of a colloidal crystal of TiO_2_@Pt MMs under the superimposed UV_YZ_ and UV_Z_ irradiation.

Movie S6.

Dynamic phase transitions of a “phototactic colloidal crystal” by alternatively turning the constituent UV lights of the superimposed irradiation on and off.

Movie S7.

On-the-fly phase transitions of the “phototactic colloidal crystals” by adjusting the light intensity of the superimposed UV lights.

Movie S8.

“Trimming” an irregular colloidal crystal into a rectangular pattern under the superimposed irradiation consisting of a global UV_YZ_ and a local rectangular UV_Z_.

Movie S9.

“Trimming” a large rectangular colloidal crystal into a circular one under the superimposed irradiation consisting of a global UV_XZ_ and a local circular UV_Z_.
